# Supplementary material for: Mechanistic analysis of enhancer sequences in the estrogen receptor transcriptional program
Source: Commun Biol. 2024 Jun 11;7:719. doi: 10.1038/s42003-024-06400-5 (PMC11167054; doi:10.1038/s42003-024-06400-5)
Supplement: Supplementary file 2 — Supplementary Information [file 42003_2024_6400_MOESM2_ESM.pdf]

Supplementary information for: **Mechanistic analysis of enhancer sequences in the Estrogen Receptor transcriptional program**

Supplementary Table 1. List of RF identified important features.

RF important Features
ERα
JUN-1
YBX1
LEF1
NKX3-1
FOXA1
RARα
NR5A2
YBX1:ER
RELA
YBX1:RUNX1
PBX1
PGR
PAX2
NR3C1
RUNX1

Supplementary Table 2. Full table of Literature evidence supporting the roles identified by GEMSTAT models. "% enhancer affected" indicates the percent of enhancers affected by ≥ 5 percentile (ensemble average). "Avg. % change" represents the predicted percentile change in activity after knock down, averaged over enhancers that were affected more than 5 percentile points.

TF	GEMSTAT inferred role	% enhancer affected	Avg. % change	Literature-suggested role for TF	Reference PMID
ERα	Strong activator	63.8	-32.5	Transcriptional activator	26884552
FOXA1	Weak activator	47.1	-20.0	Pioneer factor. Alters DNA accessibility profile and is necessary for	18358809, 16009131, 21151129
GATA3	Weak activator	2.3	-24.6	Pioneer factor	23172872, 32232341
LEF1	Weak inhibitor	2.0	13.1	Represses ERα activity by competitively binding to chromatin/recruiting HDAC1	18794125
NKX3-1	Weak inhibitor	22.4	16.0	Represses ERα activity by competitively binding to chromatin/recruiting HDAC1	18794125
GR	Strong/Weak inhibitor	0.7	18.1	Unclear; Co-activation of ERα and GR leads to altered ER binding landscape	23803465
NR5A2	Strong activator	26.7	-20.2	Activates ERα-mediated transcription at least partly through co-binding to Estrogen Response Elements.	22359603
PAX2	Strong activator	7.45	-24.13	Upregulated in ER+ breast cancer	19005469,22 168360
PBX1	Weak activator	6.4	-24.7	Pioneer factor	22125492
PGR	Strong/Weak inhibitor	6.4	-9.2	Unclear/controversial	26153859, 28729413,
RARα	Strong Activator	3.3	-21.9	Unclear/controversial	19563758, 20080953,
RELA (NFκb)	Ambiguous (weak activator/strong inhibitor)	9.3	-13.1	Transcriptional activation; controversial since ERα is known to repress NFκb transcriptional activity	25752574 ,20705611 ,19920189,1 8703630
RUNX1	Weak activator	10.55	-6.75	Mediates indirect binding (tethered) of ER to DNA	20547749
SP1	Weak activator	18.8	-8.7	Transcriptional activation in part due to mediation of indirect ERα-DNA	9328340,112 50935,11345 900
AP2-γ	Strong Activator	9.0	-12.8	Pioneer factor	21572391
YBX1	Weak/Strong inhibitor	9.8	10.1	Transcriptional repression through direct interaction with ERα	29180470

ERα:FOXA1	Cooperative	17.8	-17.7	Cooperative activity essential for ERα activity	18358809, 16009131 [illegible]
ERα:GATA3	Cooperative	3.1	-28.1	Cooperative activity as a [illegible]	23172872, [illegible]
ERα:PBX1	Cooperative	0.3	-10.1	Pioneer factor	22125492
ER:PGR	Ambiguous	0.57	-11.87	Controversial	26153859,28729413,27885264
ERα:RARα	Cooperative	1.7	-17.8	Controversial	19563758,20080953,
ERα: AP2-γ	Cooperative	1.3	-8.9	Pioneer factor	21572391
ERα:YBX1	Competitive	0.6	7.3	Transcriptional repression through direct interaction with ER	29180470

Supplementary Table 3. List of TFs identified through literature survey and the PubMed ID of their corresponding reference.

Transcription Factor	PMID
ERα	26884552
FOXA1	16009131, 26884552, 27791031, 21151129
GATA3	23172872, 21878914
NFIB	29180470
YBX1	29180470
AR	27565181
GR	29279606
PGR	28729413, 29435103
PBX1	22125492
AP-1	19339991, 26906743
AP-2γ	21572391
SP1	15695368
NFκb	25752574
CEBP	25752574
RARγ	20080953, 28977594, 21940749
RXR	21940749
NKX3-1	18794125
LEF-1	18794125
NR5A2 (LRH-1)	24049078
RUNX1	20547749
OCT4 (POU5F1)	27065334
MYC	21779462
MAX	21779462
XBP1	21297881
PPARG	23375374
PPARD	23375374
RARα	20080953, 19563758
RXRβ	23375374
NR2F2	26894976
NR2C1	28087820

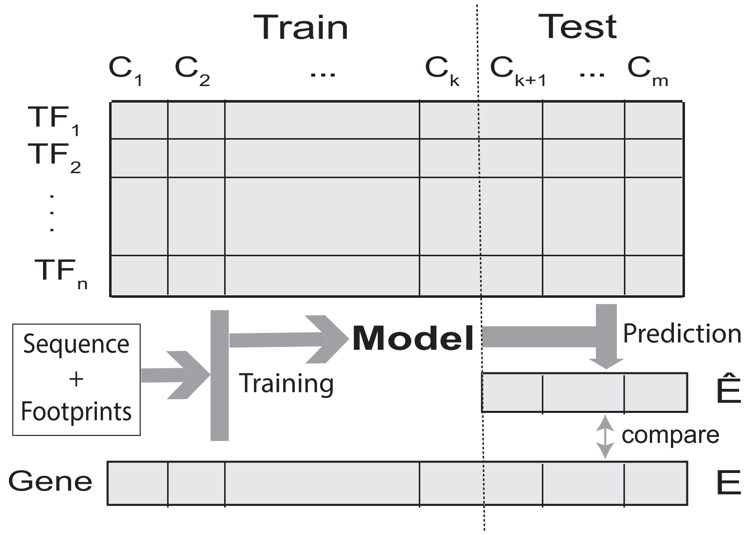

Supplementary Figure 1. Seq2expr Gene expression modeling framework.

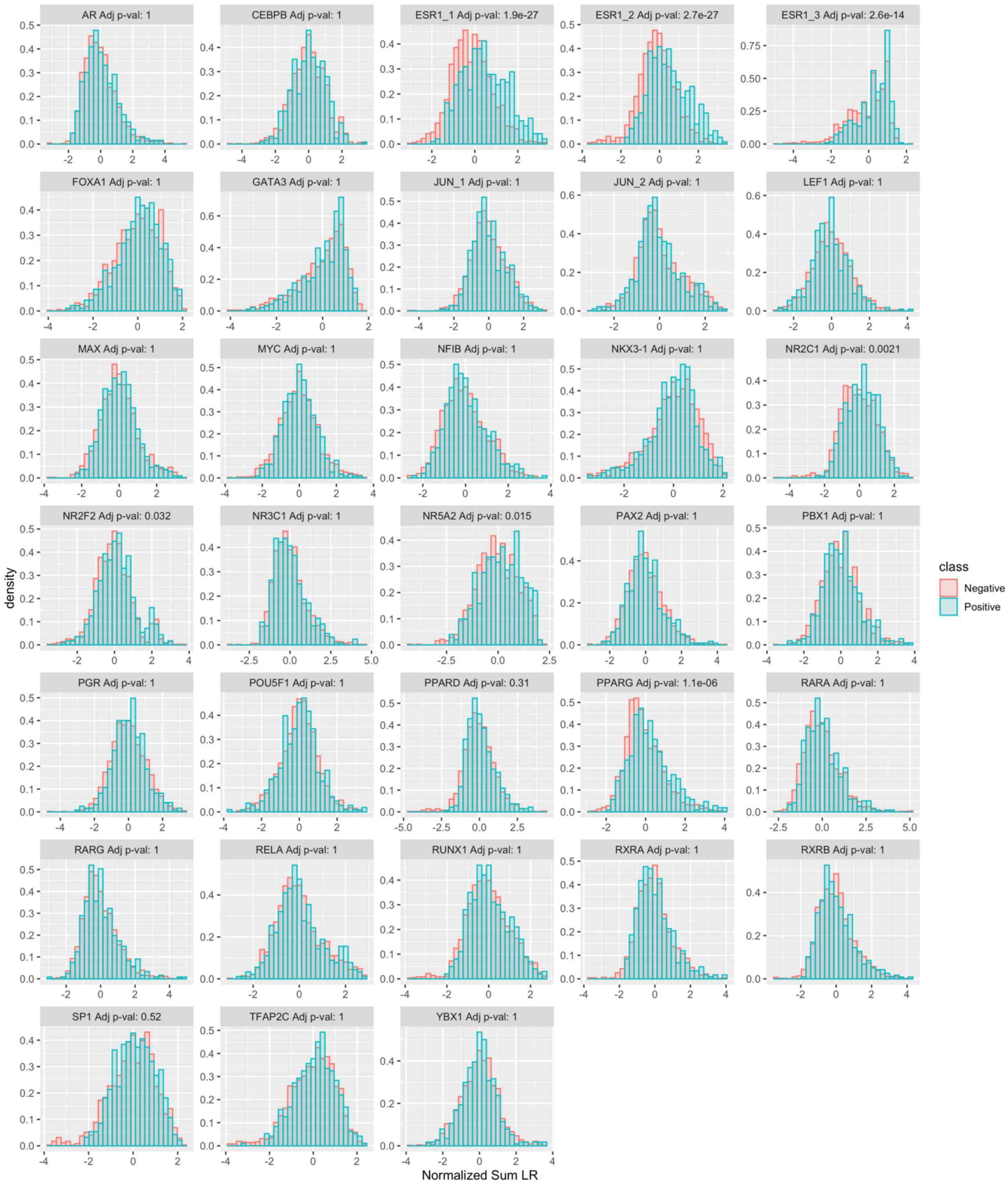

Supplementary Figure 2. Comparison of normalized predicted affinity score for each TF between positive and negative class enhancers. Each panel illustrates overlapping histogram of log-normalized sum of likelihood ratios. Bonferroni adjusted t-test p-values is reported for each panel.

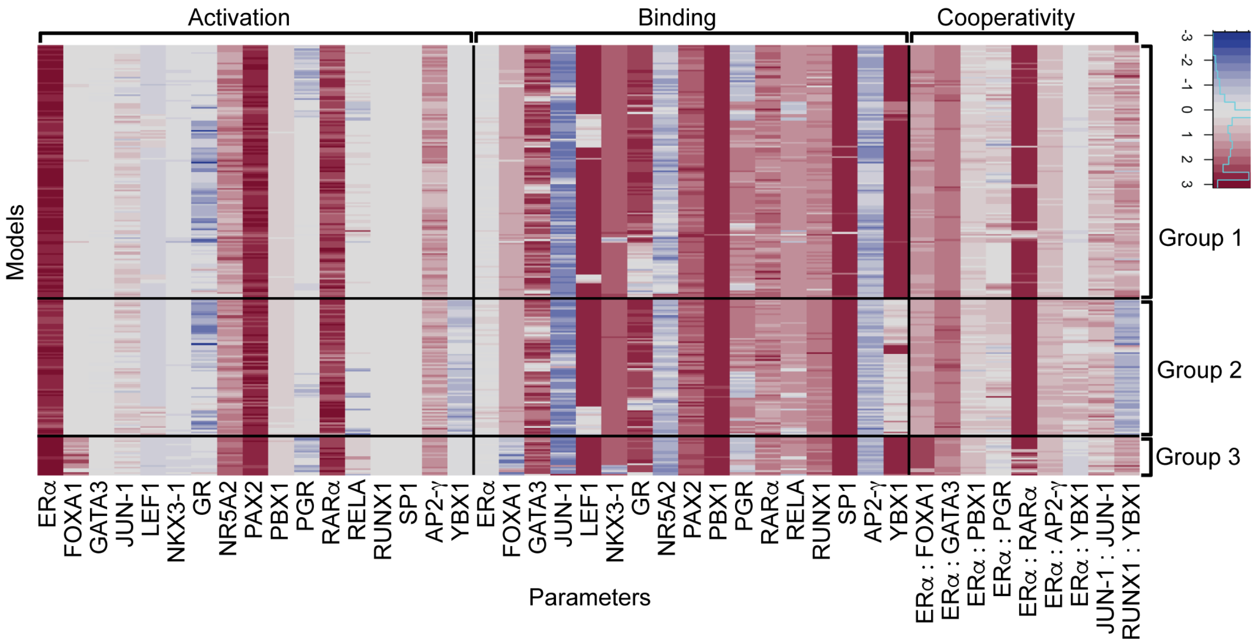

Supplementary Figure 3. Full representation of model parameters for the ensemble of GEMSTAT models. This figure is a complete version of figure 3f in the main text.

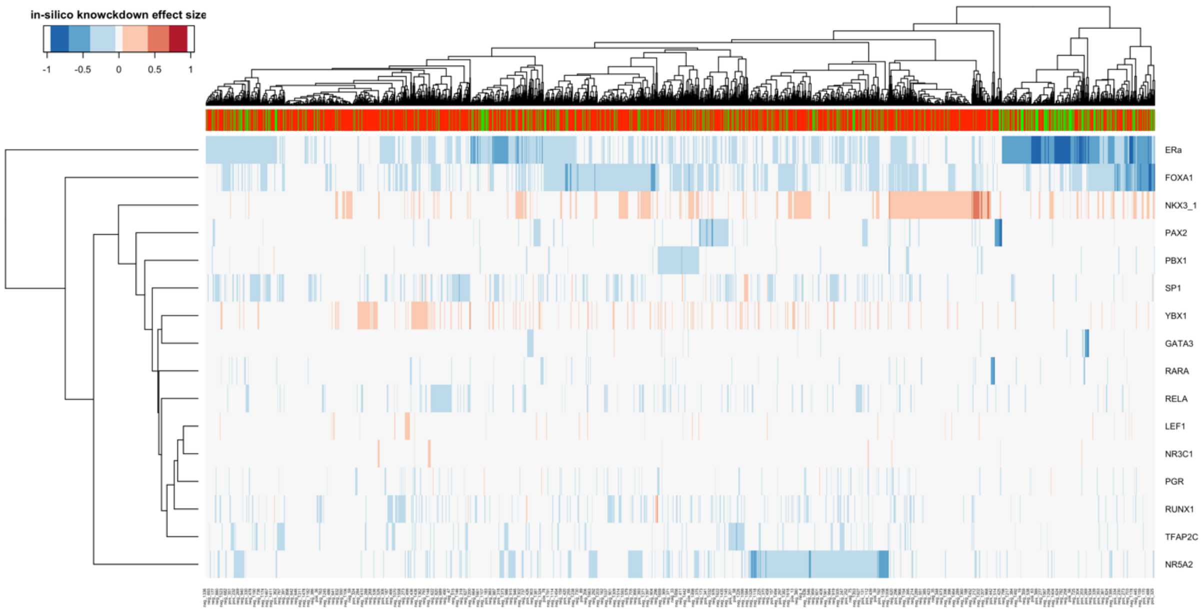

Supplementary Figure 4. TF removal effect on individual enhancers. The heatmap illustrates the average percent change in predicted expression of the examined enhancers (columns), after removal of each considered TF (rows). Red and green column side color bars show negative and positive class enhancers, respectively.

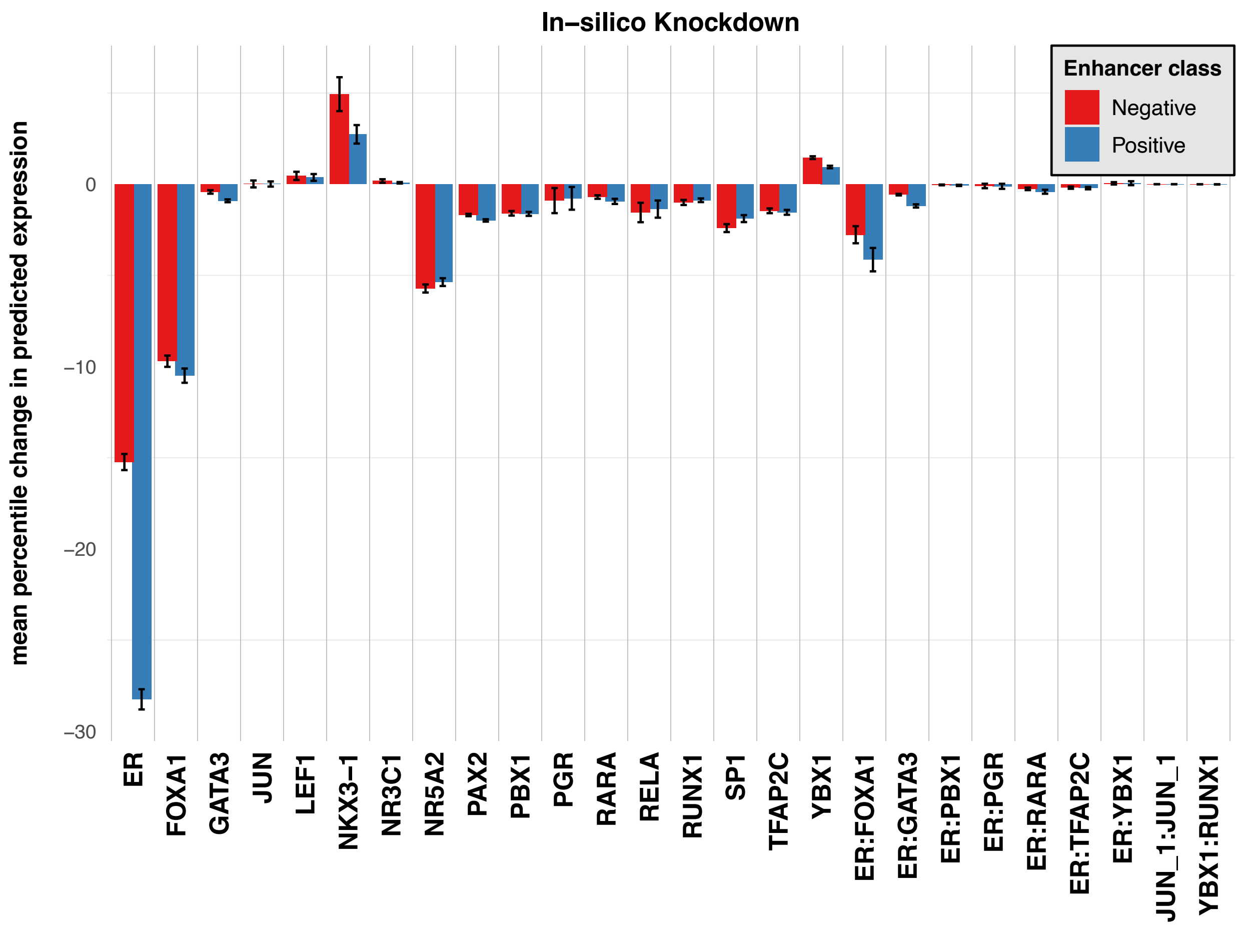

Supplementary Figure 5. In-Silico perturbation of top-ranking ensemble models. Bars represent the perturbation effect of TFs or TF-TF interactions considered by the models, separated by their affecting enhancer class. Positive and Negative classes refer to eRNA expressing and silent enhancers, respectively. Bar height represents the average over 244 top ranking models, and error bars represent the standard deviation. Y-axis indicates the average percentile change in predicted expression due to each trans perturbation.

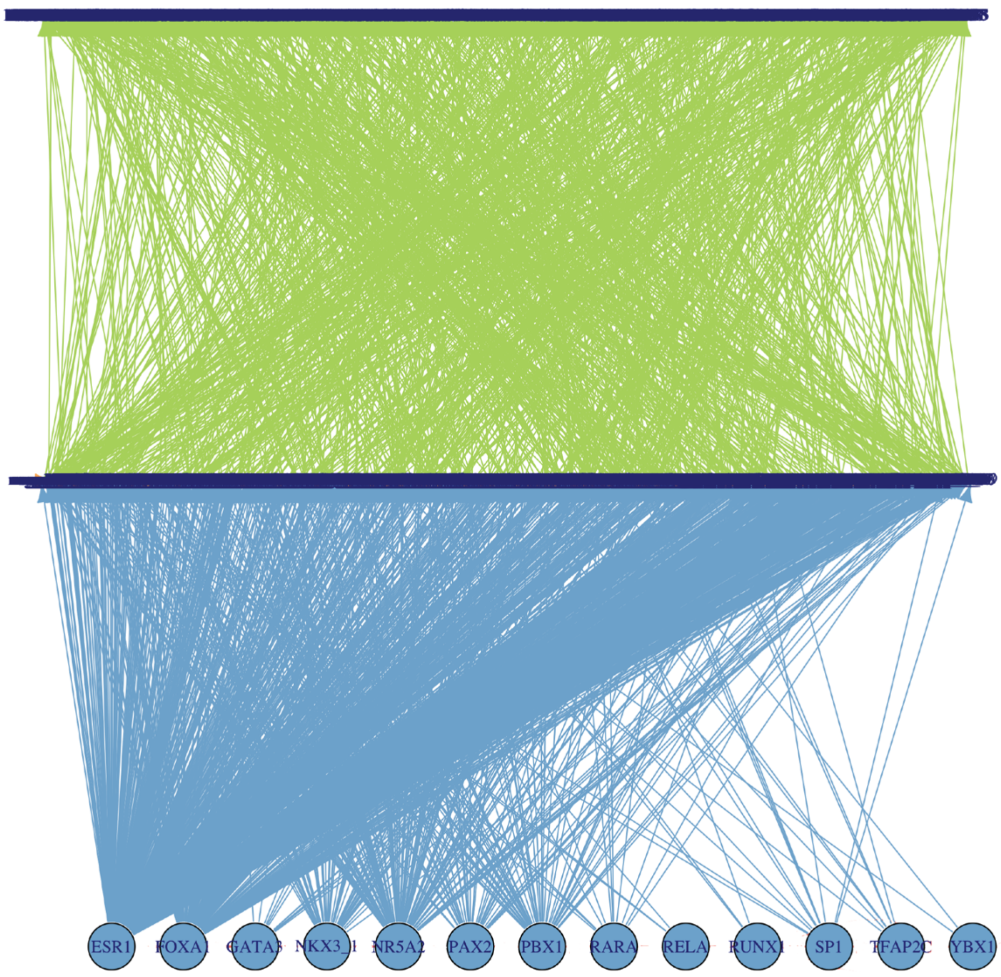

Supplementary Figure 6. Overview of TF-enhancer-gene network. Bottom, middle, and top layers of the network represent TFs, enhancers, and genes, respectively. TF to enhancer edges were derived from in-silico knockdown experiments (Enhancers affected by more than 0.3 percentile levels after a TF knockdown were marked as that TF's targets.). Enhancer to gene edges represent genomic proximity (+/- 10kb) or interaction (downloaded from 4Dgenome specific to MCF-7 cell line).

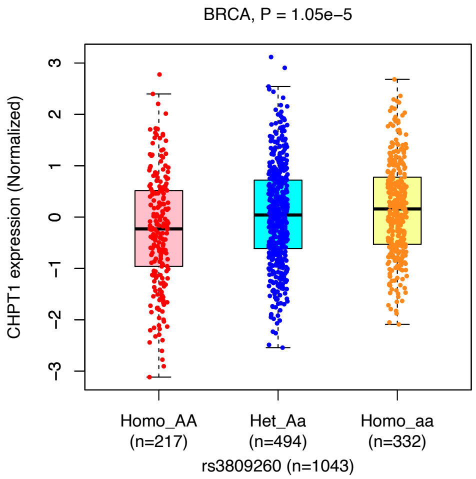

Supplementary Figure 7. Expression profile for gene CHPT1 stratified by rs3809260 variant in BRCA patients. Figure obtained from PancanQTL.

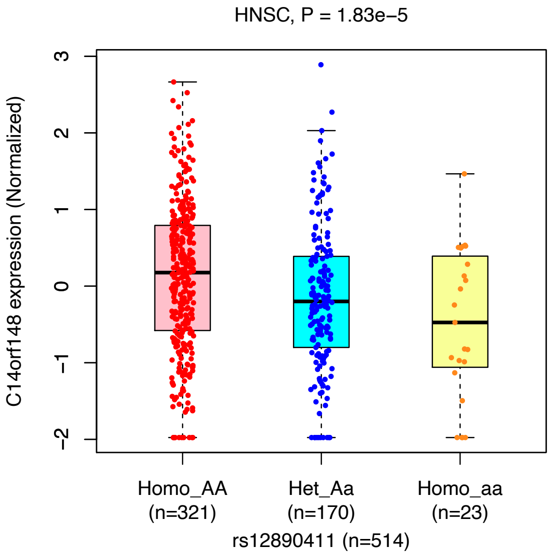

Supplementary Figure 8. Expression profile for gene NOXRED1 stratified by rs12890411 variant in HNSC patients. Figure obtained from PancanQTL.

Supplementary Note 1

Genetic variants used in this study were obtained from the following sources:

(1) Breast/Mammary tissue GTEX eQTLs downloaded from https://storage.googleapis.com/gtex_analysis_v8/single_tissue_qtl_data/GTEx_Analysis_v8_eQTL.tar],
(2) Pancan BRCA cis and trans eQTLs downloaded from http://gong_lab.hzau.edu.cn/PancanQTL/static/download/BRCA_tumor.cis_eQTL.xls and http://gong_lab.hzau.edu.cn/PancanQTL/static/download/BRCA_tumor.trans_eQTL.xls
(3) COSMIC breast cancer non-coding variants downloaded from https://cancer.sanger.ac.uk/cosmic/download and filtered by cancer type: "breast"
(4) Common SNPs are obtained from dbSNP build 151, subject to a minor allele frequency threshold of greater than 1%. [downloaded from https://ftp.ncbi.nih.gov/snp/organisms/human_9606_b151_GRCh38p7/VCF/00-common_all.vcf.gz].

Supplementary Note 2

A grid search was performed to identify optimal annotation threshold parameter among three candidates for each TF. In this exercise we trained randomly initialized GEMSTAT ensembles with three different fixed annotation thresholds for each TF, one by one (17 *3 ensembles). The validation performance of GEMSTAT models were used to identify the optimal annotation threshold parameter for each TF. In this exercise we obtained annotation threshold for each of the 17 considered TFs. Additionally, parameter sets from models with best validation performance in this grid search exercise were used as initialization points for the final GEMSTAT ensemble run with optimized annotation thresholds.
