# Supplementary material for: Mechanistic analysis of enhancer sequences in the estrogen receptor transcriptional program
Source: Commun Biol. 2024 Jun 11;7:719. doi: 10.1038/s42003-024-06400-5 (PMC11167054; doi:10.1038/s42003-024-06400-5)
Supplement: Supplementary file 3 — Description of Additional Supplementary Files [file 42003_2024_6400_MOESM3_ESM.pdf]

Description of Additional Supplementary Files

File name: Supplementary Data 1

Description: contains the coordinates of positive and negative group enhancers, as well as coordinates of all the eQTL, common, and somatic variants overlapping with 2118 enhancers of interest in this study in hg38 genome version.

File name: Supplementary Data 2

Description: contains the average predicted effect of each considered TF on studied enhancers. Each row represents an enhancer, and each column represents a TF.

File name: Supplementary Data 3

Description: contains the sequence of the six tested enhancers (WT and variants), and their associated constructs.

File name: Supplementary Data 4

Description: contains processed experimental data from luciferase assays. Dual luciferase reporter assay detected differential activity of the enhancer constructs with or without E2.

File name: Supplementary Data 5

Description: contains unprocessed readings from dual luciferase reporter assays. Each sample was measured in duplicates to calculate the firefly and Renilla luciferase signals. Firefly luciferase activity was measured first, followed by Renilla based on the manufacturer protocol.

File name: Supplementary Data 6

Description: The source data behind the graphs in the paper
